# 8-(2,2,2-Trifluoro­ethoxy)quinolinium perchlorate–8-(2,2,2-trifluoro­ethoxy)quinoline (1/1)

**DOI:** 10.1107/S1600536811013250

**Published:** 2011-04-13

**Authors:** Jun Wu, Lu-Sheng Chen, Qi-Kui Liu, Dian-Shun Guo

**Affiliations:** a Department of Chemistry, Shandong Normal University, Jinan 250014, People’s Republic of China

## Abstract

The title compound, C_11_H_9_F_3_NO^+^·ClO_4_^−^·C_11_H_8_F_3_NO or [(C_11_H_8_F_3_NO)H(C_11_H_8_F_3_NO)]ClO_4_, contains two 8-(2,2,2-trifluoro­eth­­oxy)quinoline molecules, one of which combines a proton from perchloric acid to form the corresponding quinolinium cation. The quinolinium and quinoline rings form a cationic unit *via* an inter­molecular N—H⋯N hydrogen bond. The heterocyclic units are almost perpendicular to each other [inter­planar angle 86.97 (6)°]. In the crystal, each perchlorate anion bridges two adjacent cationic units and creates a chain by a combination of C—H⋯O hydrogen bonds. Two inversion-related chains associate into a mol­ecular column by π–π stacking inter­actions between the quinolinium rings. The perpendicular and centroid–centroid distances between adjacent quinolinium rings are 3.501 (3) and 3.634 (9) Å, respectively. The molecular column is linked to its neighbors, creating a two-dimensional network *via* the weak π–π stacking between the quinoline rings [perpendicular and centroid–centroid separations 3.340 (4) and 4.408 (4) Å, respectively]. Finally, a three-dimensional framework is formed by a combination of intermolecular C—F⋯π contacts. One –CF_3_ group is disordered over two positions of equal occupancy.

## Related literature

For background to quinoline derivatives, see: Moret *et al.* (2006 ▶); Kalita *et al.* (2009 ▶). For related structures, see: Ouyang & Khoo *et al.* (1998 ▶); Karmakar *et al.* (2009 ▶); Al-Mandhary & Steel (2003 ▶); Zhang *et al.* (2006 ▶); Zheng *et al.* (2006 ▶). For π–π stacking, see: Kalita & Baruah (2010 ▶); Chen *et al.* (2005 ▶); Liang *et al.* (2002 ▶). For C—F⋯π contacts, see: Prasanna & Row (2000 ▶); Saraogi *et al.* (2003 ▶); Choudhury & Row (2004 ▶). 
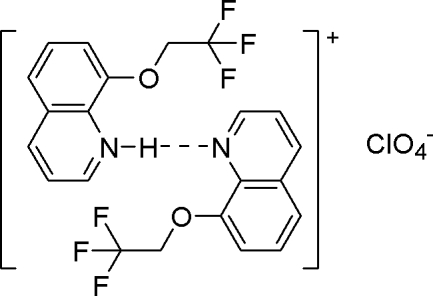


## Experimental

### 

#### Crystal data

C_11_H_9_F_3_NO^+^·ClO_4_^−^·C_11_H_8_F_3_NO
*M* *_r_* = 554.83
Triclinic, 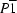

*a* = 9.462 (2) Å
*b* = 11.229 (3) Å
*c* = 11.832 (3) Å
α = 82.910 (3)°
β = 77.048 (3)°
γ = 74.536 (3)°
*V* = 1178.0 (5) Å^3^
*Z* = 2
Mo *K*α radiation
μ = 0.25 mm^−1^
*T* = 293 K
0.50 × 0.32 × 0.25 mm

#### Data collection

Bruker SMART CCD area-detector diffractometer
Absorption correction: multi-scan (*SADABS*; Bruker, 1999 ▶) *T*_min_ = 0.884, *T*_max_ = 0.940
6301 measured reflections
4364 independent reflections
3400 reflections with *I* > 2σ(*I*)
*R*_int_ = 0.021

#### Refinement

*R*[*F* ^2^ > 2σ(*F* ^2^)] = 0.050
*wR*(*F* ^2^) = 0.137
*S* = 1.02
4364 reflections
361 parameters
H-atom parameters constrained
Δρ_max_ = 0.25 e Å^−3^
Δρ_min_ = −0.27 e Å^−3^

### 

Data collection: *SMART* (Bruker, 1999 ▶); cell refinement: *SAINT* (Bruker, 1999 ▶); data reduction: *SAINT*; program(s) used to solve structure: *SHELXS97* (Sheldrick, 2008 ▶); program(s) used to refine structure: *SHELXL97* (Sheldrick, 2008 ▶); molecular graphics: *SHELXTL* (Sheldrick, 2008 ▶); software used to prepare material for publication: *SHELXTL*.

## Supplementary Material

Crystal structure: contains datablocks I, global. DOI: 10.1107/S1600536811013250/im2278sup1.cif

Structure factors: contains datablocks I. DOI: 10.1107/S1600536811013250/im2278Isup2.hkl

Additional supplementary materials:  crystallographic information; 3D view; checkCIF report

## Figures and Tables

**Table 1:** Hydrogen-bond geometry (Å, °) *Cg*1, *Cg*2 and *Cg*3 are the midpoints of the N1–C5, C5–C9 and C17–C18 bonds, respectively.

*D*—H⋯*A*	*D*—H	H⋯*A*	*D*⋯*A*	*D*—H⋯*A*
N1—H1*A*⋯N2	0.86	1.87	2.684 (3)	158
C22—F5⋯*Cg*1	1.33	3.10	3.796 (3)	111
C22—F6⋯*Cg*2	1.33	3.17	3.804 (4)	109
C13—H13⋯O3^i^	0.93	2.60	3.393 (6)	144
C21—H21*B*⋯O4^ii^	0.97	2.48	3.437 (7)	169
C22—F5⋯*Cg*3^iii^	1.33	3.24	3.860 (8)	108

Symmetry codes: (i) 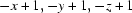; (ii) 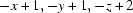; (iii) 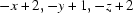.

**Fig. 1.**
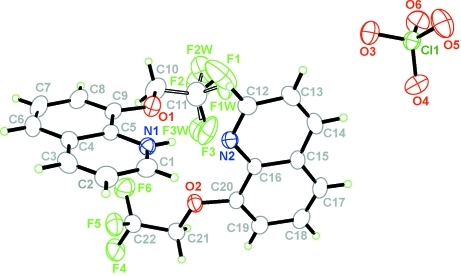
Molecular structure of the title complex, with displacement ellipsoids drawn at the 30% probability level.

**Fig. 2.**
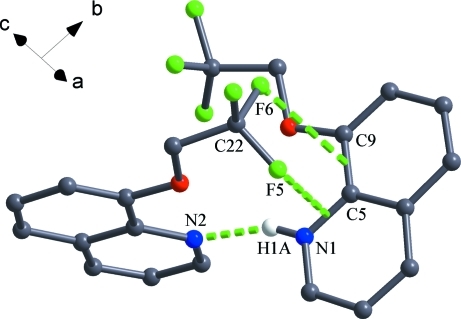
Hydrogen-bonded cationic unit, with weak C—F···π contacts. For the sake of clarity, the disordered moieties and the H atoms not involved in hydrogen bonding have been omitted.

**Fig. 3.**
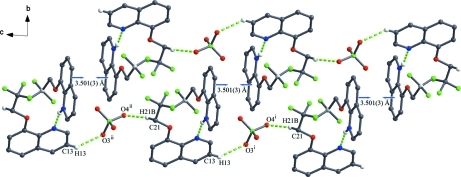
Molecular column formed by hydrogen bonds and π–π stackings, viewed along the a axis. The disordered moieties and H atoms not involved in hydrogen bonding have been omitted for clarity. [Symmetry codes: (i) -x + 1, -y + 1, -z + 1; (ii) -x + 1, -y + 1, -z + 2].

## supplementary crystallographic information

### Comment

Molecules containing quinoline moieties have attracted much interest due to their significant bioactivities (Moret *et al.*, 2006) and potential applications for constructing supramolecular systems by various hydrogen bonds and π–π interactions (Kalita *et al.*, 2009). Numerous crystal structures of 8-substituented quinolines, most of which are 8-alkyloxyquinoline derivatives have been reported (Karmakar *et al.*, 2009; Al-Mandhary *et al.*, 2003; Zhang *et al.*, 2006; Zheng *et al.*, 2006), while the crystal structures of such compounds exhibiting fluorinated alkyl groups have rarely been described. We synthesized a new trifluoroethoxyquinoline derivative and attempted to prepare its Ni(II)-complex. Surprisingly, a complex without the Ni(II) ion, namely 8-(2,2,2-trifluoroethoxy)-quinolinium-[8-(2,2,2-trifluoroethoxy)-quinoline] perchlorate, was obtained. Here, we report its crystal structure.

In the crystal structure of the title complex the asymmetric unit contains one perchlorate anion and one organic cation consisting of two 8-(2,2,2-trifluoroethoxy)-quinolines and a proton (Fig. 1). In the cationic unit, one quinoline ring is protonated forming a quinolinium and is connected to the other one *via* a N—H···N hydrogen bond (N–H···N angle 155.33 (5)°, N1···N2 2.684 (3) Å, Fig. 2, Table 1). This distance is shorter than 2.729 Å reported for a similar quinoline derivative (Ouyang *et al.*, 1998). Such a short distance may be ascribed to the presence of two weak C—F···π contacts (Prasanna & Row, 2000; Saraogi *et al.*, 2003; Choudhury & Row, 2004) between the non-disordered trifluoromethyl group and the quinoline ring. Separations of F5···*Cg*1 and F6···*Cg*2 (*Cg*1 and *Cg*2 are the centroids of N1–C5 and C5–C9 bonds, respectively) are 3.099 (3) and 3.166 (3) Å, respectively. The quinolinium and quinoline rings are almost perpendicular to each other, with a dihedral angle of 86.97 (6)°. The other trifluoromethyl group is disordered over two orientations, with refined site-occupancy factors of 0.5.

In the packing of the title complex, there exist intermolecular C—H···O hydrogen bonds, C—F···π contacts (Table 1) and π–π stackings (Kalita & Baruah, 2010; Chen *et al.*, 2005; Liang *et al.*, 2002). The cationic units are alternately bridged by perchlorate anions with intermolecular C—H···O hydrogen bonds and form an infinite one-dimensional chain along the *c* axis (Fig. 3). Hydrogen bonds arise from atoms C13—H13 and C21—H21B in the molecule at (*x*, *y*, *z*) acting as hydrogen bond donors towards atoms O3 at (-*x* + 1, -*y* + 1, -*z* + 1) and O4 (-*x* + 1, -*y* + 1, -*z* + 2), respectively. Two adjacent chains running along the alternate orientation are further combined to a molecular column by π–π stacking interactions between the quinolinium rings, with perpendicular and centroid-centroid distances of 3.501 (3) and 3.634 (9) Å, respectively, between neighboring phenyl rings of the quinolinium units. Finally, a complicated three-dimensional framework is formed by a combination of C—F···π contacts, with a F5···*Cg*3 (*Cg*3 is the centroid of C17–C18 bond) distance of 3.240 (7) Å (Table 1), and weak π–π stackings between adjacent phenyl rings of the quinoline moieties, with perpendicular and centroid-centroid separations of 3.340 (4) and 4.408 (4) Å, respectively.

### Experimental

A suspension of 8-hydroxyquinoline (0.200 g, 1.378 mmol), anhydrous KOH (0.093 g, 1.657 mmol) and 2,2,2-trifluoroethyl-4-methylbenzenesulfonate (0.385 g, 1.514 mmol) in dry NMP (5 ml) was stirred for 5 h at 393 K and then cooled to room temperature. The resulting mixture was neutralized with 5% aqueous HCl and extracted with CH_2_Cl_2_. The organic layer was separated and washed with saturated sodium hydrogen carbonate and brine, and dried over anhydrous MgSO_4_. Removal of the solvent under reduced pressure gave 8-(2,2,2-trifluoroethoxy)-quinoline as a yellow solid (yield 73%), which was purified by flash column chromatography (EtOAc/petroleum ether = 1:3, *R*_F_ = 1/2).

Single crystals of the title complex suitable for X-ray analysis were obtained from a solution of Ni(ClO_4_)_2_ (0.013 g, 0.050 mmol) in MeOH (1 ml) which was layered onto a solution of 8-(2,2,2-trifluoroethoxy)-quinoline (0.012 g, 0.053 mmol) in CH_2_Cl_2_ (1 ml) at 298 K.

### Refinement

All non-hydrogen atoms were refined with anisotropic displacement parameters. Hydrogen atoms attached to anisotropically refined atoms were placed in geometrically idealized positions and included as riding atoms with C—H = 0.93Å and *U*_iso_(H) = 1.2*U*_eq_(C) (aromatic); C—H = 0.97Å and *U*_iso_(H) = 1.2*U*_eq_(C) (methylene); N—H = 0.86Å and *U*_iso_(H) = 1.2*U*_eq_(N). In the title molecule, one trifluoromethyl group was disordered over two orientations, with refined site occupation factors of 0.5: 0.5.

### Crystal data

**Table:**

C_11_H_9_F_3_NO^+^·ClO_4_^−^·C_11_H_8_F_3_NO	*Z* = 2
*M**_r_* = 554.83	*F*(000) = 564
Triclinic, *P*1	*D*_x_ = 1.564 Mg m^−^^3^
Hall symbol: -P 1	Mo *K*α radiation, λ = 0.71073 Å
*a* = 9.462 (2) Å	Cell parameters from 2404 reflections
*b* = 11.229 (3) Å	θ = 2.5–25.1°
*c* = 11.832 (3) Å	µ = 0.25 mm^−^^1^
α = 82.910 (3)°	*T* = 293 K
β = 77.048 (3)°	Block, colourless
γ = 74.536 (3)°	0.50 × 0.32 × 0.25 mm
*V* = 1178.0 (5) Å^3^	

### Data collection

**Table:**

Bruker SMART CCD area-detector diffractometer	4364 independent reflections
Radiation source: fine-focus sealed tube	3400 reflections with *I* > 2σ(*I*)
graphite	*R*_int_ = 0.021
phi and ω scans	θ_max_ = 25.6°, θ_min_ = 1.8°
Absorption correction: multi-scan (*SADABS*; Bruker, 1999)	*h* = −11→8
*T*_min_ = 0.884, *T*_max_ = 0.940	*k* = −13→9
6301 measured reflections	*l* = −14→14

### Refinement

**Table:**

Refinement on *F*^2^	Primary atom site location: structure-invariant direct methods
Least-squares matrix: full	Secondary atom site location: difference Fourier map
*R*[*F*^2^ > 2σ(*F*^2^)] = 0.050	Hydrogen site location: inferred from neighbouring sites
*wR*(*F*^2^) = 0.137	H-atom parameters constrained
*S* = 1.02	*w* = 1/[σ^2^(*F*_o_^2^) + (0.068*P*)^2^ + 0.293*P*] where *P* = (*F*_o_^2^ + 2*F*_c_^2^)/3
4364 reflections	(Δ/σ)_max_ < 0.001
361 parameters	Δρ_max_ = 0.25 e Å^−^^3^
0 restraints	Δρ_min_ = −0.27 e Å^−^^3^

### Special details

**Table:**

Experimental. ^1Ĥ NMR (300 MHz, CDCl~3~):*δ* 8.97 (*dd*, 1H, *J* = 4.04 Hz, 1.52 Hz), 8.17 (*d*, 1H, *J* = 7.52 Hz), 7.54–7.44 (*m*, 3H), 7.25 (*d*, H, *J* = 7.65 Hz), 4.78 (*q*, 2H, *J* = 8.33 Hz).
Geometry. All e.s.d.'s (except the e.s.d. in the dihedral angle between two l.s. planes) are estimated using the full covariance matrix. The cell e.s.d.'s are taken into account individually in the estimation of e.s.d.'s in distances, angles and torsion angles; correlations between e.s.d.'s in cell parameters are only used when they are defined by crystal symmetry. An approximate (isotropic) treatment of cell e.s.d.'s is used for estimating e.s.d.'s involving l.s. planes.
Refinement. Refinement of *F*^2^ against ALL reflections. The weighted *R*-factor *wR* and goodness of fit *S* are based on *F*^2^, conventional *R*-factors *R* are based on *F*, with *F* set to zero for negative *F*^2^. The threshold expression of *F*^2^ > σ(*F*^2^) is used only for calculating *R*-factors(gt) *etc*. and is not relevant to the choice of reflections for refinement. *R*-factors based on *F*^2^ are statistically about twice as large as those based on *F*, and *R*- factors based on ALL data will be even larger.

### Fractional atomic coordinates and isotropic or equivalent isotropic displacement parameters (Å^2^)

**Table:**

	*x*	*y*	*z*	*U*_iso_*/*U*_eq_	Occ. (<1)
C1	1.1468 (3)	0.6885 (3)	0.6473 (2)	0.0583 (6)	
H1	1.1569	0.6034	0.6558	0.070*	
C2	1.2727 (3)	0.7332 (3)	0.6049 (2)	0.0694 (8)	
H2	1.3662	0.6791	0.5845	0.083*	
C3	1.2575 (3)	0.8571 (3)	0.5937 (2)	0.0682 (8)	
H3	1.3416	0.8880	0.5653	0.082*	
C4	1.1164 (3)	0.9399 (2)	0.6243 (2)	0.0548 (6)	
C5	0.9930 (3)	0.8878 (2)	0.66440 (18)	0.0448 (5)	
C6	1.0927 (4)	1.0696 (3)	0.6165 (2)	0.0677 (8)	
H6	1.1731	1.1056	0.5909	0.081*	
C7	0.9530 (4)	1.1412 (3)	0.6463 (2)	0.0738 (8)	
H7	0.9385	1.2268	0.6415	0.089*	
C8	0.8288 (3)	1.0905 (2)	0.6843 (2)	0.0643 (7)	
H8	0.7334	1.1424	0.7027	0.077*	
C9	0.8474 (3)	0.9661 (2)	0.6943 (2)	0.0505 (6)	
C10	0.5911 (3)	0.9754 (3)	0.7691 (3)	0.0844 (10)	
H10A	0.5518	1.0228	0.7037	0.101*	
H10B	0.5902	1.0325	0.8245	0.101*	
C12	0.7912 (3)	0.5853 (2)	0.6481 (2)	0.0637 (7)	
H12	0.8219	0.6259	0.5770	0.076*	
C13	0.7078 (4)	0.4983 (3)	0.6515 (3)	0.0731 (8)	
H13	0.6806	0.4839	0.5845	0.088*	
C14	0.6678 (3)	0.4362 (2)	0.7528 (3)	0.0687 (8)	
H14	0.6140	0.3772	0.7557	0.082*	
C15	0.7062 (3)	0.4590 (2)	0.8546 (2)	0.0533 (6)	
C16	0.7845 (2)	0.55191 (19)	0.8454 (2)	0.0449 (5)	
C17	0.6663 (3)	0.3978 (2)	0.9643 (3)	0.0664 (7)	
H17	0.6164	0.3355	0.9710	0.080*	
C18	0.7003 (3)	0.4296 (2)	1.0592 (3)	0.0670 (7)	
H18	0.6739	0.3884	1.1308	0.080*	
C19	0.7750 (3)	0.5238 (2)	1.0520 (2)	0.0574 (6)	
H19	0.7962	0.5450	1.1188	0.069*	
C20	0.8163 (2)	0.5841 (2)	0.9478 (2)	0.0465 (5)	
C21	0.9028 (3)	0.7245 (2)	1.0323 (2)	0.0550 (6)	
H21A	0.9774	0.6653	1.0687	0.066*	
H21B	0.8083	0.7397	1.0876	0.066*	
C22	0.9496 (3)	0.8415 (3)	0.9960 (2)	0.0621 (7)	
Cl1	0.35067 (7)	0.30823 (6)	0.64594 (5)	0.0564 (2)	
F4	0.9743 (3)	0.88475 (17)	1.08657 (15)	0.0965 (6)	
F5	1.07304 (19)	0.82890 (16)	0.91311 (15)	0.0783 (5)	
F6	0.8460 (2)	0.92888 (15)	0.95375 (16)	0.0850 (5)	
N1	1.0134 (2)	0.76298 (17)	0.67574 (16)	0.0463 (4)	
H1A	0.9374	0.7321	0.7021	0.056*	
N2	0.8280 (2)	0.61195 (17)	0.74111 (17)	0.0482 (5)	
O1	0.73864 (18)	0.90405 (15)	0.73088 (16)	0.0613 (5)	
O2	0.88758 (18)	0.67771 (15)	0.93048 (13)	0.0526 (4)	
O3	0.4147 (3)	0.4098 (2)	0.6013 (2)	0.0932 (7)	
O4	0.4074 (2)	0.25456 (19)	0.74758 (18)	0.0835 (6)	
O5	0.3897 (3)	0.2197 (2)	0.5613 (2)	0.0979 (8)	
O6	0.1932 (2)	0.3522 (2)	0.6752 (2)	0.0888 (7)	
C11	0.4994 (4)	0.8884 (4)	0.8241 (4)	0.0902 (10)	0.50
F1	0.5026 (16)	0.8110 (15)	0.7456 (17)	0.177 (7)	0.50
F2	0.3618 (17)	0.9521 (18)	0.8772 (11)	0.131 (4)	0.50
F3	0.5473 (15)	0.8205 (11)	0.9134 (14)	0.118 (5)	0.50
C11W	0.4994 (4)	0.8884 (4)	0.8241 (4)	0.0902 (10)	0.50
F1W	0.5117 (10)	0.7933 (8)	0.7704 (12)	0.105 (4)	0.50
F2W	0.3555 (16)	0.9444 (18)	0.8260 (11)	0.109 (3)	0.50
F3W	0.5318 (19)	0.8454 (16)	0.9246 (13)	0.142 (6)	0.50

### Atomic displacement parameters (Å^2^)

**Table:**

	*U*^11^	*U*^22^	*U*^33^	*U*^12^	*U*^13^	*U*^23^
C1	0.0535 (15)	0.0587 (15)	0.0562 (15)	−0.0040 (12)	−0.0086 (12)	−0.0055 (12)
C2	0.0472 (15)	0.085 (2)	0.0672 (18)	−0.0088 (14)	−0.0025 (13)	−0.0029 (15)
C3	0.0540 (16)	0.094 (2)	0.0623 (17)	−0.0336 (15)	−0.0074 (13)	−0.0012 (15)
C4	0.0612 (16)	0.0688 (16)	0.0412 (13)	−0.0279 (13)	−0.0108 (11)	−0.0022 (11)
C5	0.0539 (13)	0.0484 (13)	0.0347 (11)	−0.0179 (11)	−0.0090 (10)	−0.0007 (9)
C6	0.087 (2)	0.0718 (18)	0.0563 (16)	−0.0452 (17)	−0.0110 (15)	0.0005 (13)
C7	0.116 (3)	0.0494 (15)	0.0600 (17)	−0.0323 (17)	−0.0124 (17)	−0.0016 (13)
C8	0.0779 (18)	0.0474 (14)	0.0599 (16)	−0.0087 (13)	−0.0078 (14)	−0.0004 (12)
C9	0.0564 (14)	0.0476 (13)	0.0445 (13)	−0.0114 (11)	−0.0081 (11)	0.0008 (10)
C10	0.0535 (17)	0.0661 (18)	0.111 (3)	0.0037 (14)	0.0015 (16)	0.0071 (17)
C12	0.0788 (19)	0.0615 (16)	0.0556 (16)	−0.0193 (14)	−0.0209 (14)	−0.0046 (12)
C13	0.085 (2)	0.0695 (18)	0.080 (2)	−0.0252 (16)	−0.0348 (17)	−0.0145 (16)
C14	0.0658 (18)	0.0522 (15)	0.099 (2)	−0.0214 (13)	−0.0283 (16)	−0.0109 (15)
C15	0.0451 (13)	0.0400 (12)	0.0764 (17)	−0.0112 (10)	−0.0144 (12)	−0.0041 (11)
C16	0.0404 (12)	0.0361 (11)	0.0566 (14)	−0.0069 (9)	−0.0101 (10)	−0.0027 (10)
C17	0.0574 (16)	0.0501 (15)	0.093 (2)	−0.0240 (12)	−0.0120 (15)	0.0066 (14)
C18	0.0654 (17)	0.0598 (16)	0.0700 (18)	−0.0220 (14)	−0.0037 (14)	0.0135 (13)
C19	0.0573 (15)	0.0566 (15)	0.0567 (15)	−0.0169 (12)	−0.0069 (12)	0.0003 (12)
C20	0.0437 (12)	0.0412 (12)	0.0534 (14)	−0.0109 (10)	−0.0062 (10)	−0.0035 (10)
C21	0.0618 (15)	0.0595 (15)	0.0467 (13)	−0.0199 (12)	−0.0097 (11)	−0.0066 (11)
C22	0.0790 (19)	0.0619 (16)	0.0541 (15)	−0.0259 (15)	−0.0184 (14)	−0.0094 (12)
Cl1	0.0547 (4)	0.0556 (4)	0.0571 (4)	−0.0178 (3)	−0.0036 (3)	−0.0017 (3)
F4	0.1570 (19)	0.0919 (12)	0.0696 (11)	−0.0634 (13)	−0.0396 (12)	−0.0119 (9)
F5	0.0817 (11)	0.0814 (11)	0.0788 (11)	−0.0408 (9)	−0.0069 (9)	−0.0032 (9)
F6	0.1038 (13)	0.0591 (10)	0.0926 (13)	−0.0115 (9)	−0.0315 (11)	−0.0039 (9)
N1	0.0444 (11)	0.0473 (11)	0.0459 (11)	−0.0131 (9)	−0.0054 (8)	−0.0011 (8)
N2	0.0519 (11)	0.0442 (10)	0.0500 (11)	−0.0117 (9)	−0.0124 (9)	−0.0051 (8)
O1	0.0449 (9)	0.0506 (10)	0.0792 (12)	−0.0075 (8)	−0.0004 (8)	−0.0011 (9)
O2	0.0648 (10)	0.0551 (9)	0.0453 (9)	−0.0284 (8)	−0.0091 (8)	−0.0050 (7)
O3	0.1065 (17)	0.0857 (15)	0.1007 (17)	−0.0546 (14)	−0.0250 (13)	0.0195 (12)
O4	0.0875 (15)	0.0769 (13)	0.0738 (13)	−0.0045 (11)	−0.0184 (11)	0.0110 (11)
O5	0.0924 (16)	0.1109 (18)	0.0948 (16)	−0.0381 (14)	0.0101 (13)	−0.0478 (14)
O6	0.0554 (12)	0.0968 (16)	0.1055 (17)	−0.0104 (11)	−0.0081 (11)	−0.0077 (13)
C11	0.0475 (18)	0.089 (3)	0.120 (4)	−0.0082 (17)	0.0047 (19)	−0.010 (3)
F1	0.109 (7)	0.217 (14)	0.235 (12)	−0.025 (7)	−0.069 (7)	−0.097 (10)
F2	0.054 (5)	0.124 (6)	0.179 (12)	0.000 (4)	0.034 (7)	−0.030 (9)
F3	0.084 (5)	0.082 (3)	0.155 (12)	−0.026 (3)	0.021 (5)	0.038 (5)
C11W	0.0475 (18)	0.089 (3)	0.120 (4)	−0.0082 (17)	0.0047 (19)	−0.010 (3)
F1W	0.056 (4)	0.077 (4)	0.181 (9)	−0.031 (3)	0.001 (4)	−0.023 (5)
F2W	0.045 (3)	0.128 (5)	0.143 (8)	−0.010 (3)	0.007 (5)	−0.041 (7)
F3W	0.142 (11)	0.194 (13)	0.093 (7)	−0.070 (9)	−0.007 (6)	0.002 (7)

### Geometric parameters (Å, °)

**Table:**

C1—N1	1.312 (3)	C14—H14	0.9300
C1—C2	1.383 (4)	C15—C16	1.413 (3)
C1—H1	0.9300	C15—C17	1.414 (4)
C2—C3	1.352 (4)	C16—N2	1.364 (3)
C2—H2	0.9300	C16—C20	1.421 (3)
C3—C4	1.408 (4)	C17—C18	1.346 (4)
C3—H3	0.9300	C17—H17	0.9300
C4—C5	1.406 (3)	C18—C19	1.404 (4)
C4—C6	1.408 (4)	C18—H18	0.9300
C5—N1	1.357 (3)	C19—C20	1.361 (3)
C5—C9	1.418 (3)	C19—H19	0.9300
C6—C7	1.346 (4)	C20—O2	1.365 (3)
C6—H6	0.9300	C21—O2	1.422 (3)
C7—C8	1.401 (4)	C21—C22	1.480 (4)
C7—H7	0.9300	C21—H21A	0.9700
C8—C9	1.354 (3)	C21—H21B	0.9700
C8—H8	0.9300	C22—F4	1.319 (3)
C9—O1	1.357 (3)	C22—F6	1.326 (3)
C10—O1	1.416 (3)	C22—F5	1.334 (3)
C10—C11	1.474 (5)	Cl1—O6	1.415 (2)
C10—H10A	0.9700	Cl1—O5	1.418 (2)
C10—H10B	0.9700	Cl1—O3	1.423 (2)
C12—N2	1.316 (3)	Cl1—O4	1.427 (2)
C12—C13	1.402 (4)	N1—H1A	0.8600
C12—H12	0.9300	C11—F3	1.317 (15)
C13—C14	1.341 (4)	C11—F1	1.338 (17)
C13—H13	0.9300	C11—F2	1.359 (15)
C14—C15	1.403 (4)		
			
N1—C1—C2	121.7 (3)	N2—C16—C15	121.5 (2)
N1—C1—H1	119.1	N2—C16—C20	119.7 (2)
C2—C1—H1	119.1	C15—C16—C20	118.8 (2)
C3—C2—C1	118.9 (3)	C18—C17—C15	120.2 (2)
C3—C2—H2	120.6	C18—C17—H17	119.9
C1—C2—H2	120.6	C15—C17—H17	119.9
C2—C3—C4	121.0 (3)	C17—C18—C19	121.3 (3)
C2—C3—H3	119.5	C17—C18—H18	119.4
C4—C3—H3	119.5	C19—C18—H18	119.4
C5—C4—C6	119.0 (3)	C20—C19—C18	120.3 (3)
C5—C4—C3	116.9 (2)	C20—C19—H19	119.8
C6—C4—C3	124.1 (3)	C18—C19—H19	119.8
N1—C5—C4	120.2 (2)	C19—C20—O2	125.5 (2)
N1—C5—C9	120.1 (2)	C19—C20—C16	120.1 (2)
C4—C5—C9	119.8 (2)	O2—C20—C16	114.40 (19)
C7—C6—C4	119.7 (3)	O2—C21—C22	107.24 (19)
C7—C6—H6	120.2	O2—C21—H21A	110.3
C4—C6—H6	120.2	C22—C21—H21A	110.3
C6—C7—C8	121.9 (3)	O2—C21—H21B	110.3
C6—C7—H7	119.0	C22—C21—H21B	110.3
C8—C7—H7	119.0	H21A—C21—H21B	108.5
C9—C8—C7	120.1 (3)	F4—C22—F6	107.2 (2)
C9—C8—H8	119.9	F4—C22—F5	108.3 (2)
C7—C8—H8	119.9	F6—C22—F5	105.6 (2)
C8—C9—O1	126.7 (2)	F4—C22—C21	109.5 (2)
C8—C9—C5	119.5 (2)	F6—C22—C21	112.6 (2)
O1—C9—C5	113.7 (2)	F5—C22—C21	113.3 (2)
O1—C10—C11	107.3 (2)	O6—Cl1—O5	109.93 (14)
O1—C10—H10A	110.3	O6—Cl1—O3	109.00 (14)
C11—C10—H10A	110.3	O5—Cl1—O3	109.86 (15)
O1—C10—H10B	110.3	O6—Cl1—O4	109.81 (14)
C11—C10—H10B	110.3	O5—Cl1—O4	110.33 (15)
H10A—C10—H10B	108.5	O3—Cl1—O4	107.87 (14)
N2—C12—C13	122.8 (3)	C1—N1—C5	121.2 (2)
N2—C12—H12	118.6	C1—N1—H1A	119.4
C13—C12—H12	118.6	C5—N1—H1A	119.4
C14—C13—C12	118.9 (3)	C12—N2—C16	118.9 (2)
C14—C13—H13	120.5	C9—O1—C10	117.2 (2)
C12—C13—H13	120.5	C20—O2—C21	116.17 (18)
C13—C14—C15	120.8 (3)	F3—C11—F1	107.5 (10)
C13—C14—H14	119.6	F3—C11—F2	100.6 (10)
C15—C14—H14	119.6	F1—C11—F2	116.5 (9)
C14—C15—C16	117.0 (2)	F3—C11—C10	113.4 (7)
C14—C15—C17	123.7 (2)	F1—C11—C10	108.9 (8)
C16—C15—C17	119.3 (2)	F2—C11—C10	109.9 (9)
			
N1—C1—C2—C3	0.6 (4)	C16—C15—C17—C18	1.2 (4)
C1—C2—C3—C4	0.0 (4)	C15—C17—C18—C19	0.3 (4)
C2—C3—C4—C5	−1.3 (4)	C17—C18—C19—C20	−0.8 (4)
C2—C3—C4—C6	179.0 (3)	C18—C19—C20—O2	179.2 (2)
C6—C4—C5—N1	−178.2 (2)	C18—C19—C20—C16	−0.2 (4)
C3—C4—C5—N1	2.1 (3)	N2—C16—C20—C19	−179.2 (2)
C6—C4—C5—C9	1.1 (3)	C15—C16—C20—C19	1.7 (3)
C3—C4—C5—C9	−178.7 (2)	N2—C16—C20—O2	1.3 (3)
C5—C4—C6—C7	−0.7 (4)	C15—C16—C20—O2	−177.81 (19)
C3—C4—C6—C7	179.0 (3)	O2—C21—C22—F4	−176.1 (2)
C4—C6—C7—C8	−0.6 (4)	O2—C21—C22—F6	64.7 (3)
C6—C7—C8—C9	1.4 (4)	O2—C21—C22—F5	−55.0 (3)
C7—C8—C9—O1	179.3 (2)	C2—C1—N1—C5	0.2 (4)
C7—C8—C9—C5	−0.9 (4)	C4—C5—N1—C1	−1.6 (3)
N1—C5—C9—C8	179.0 (2)	C9—C5—N1—C1	179.1 (2)
C4—C5—C9—C8	−0.3 (3)	C13—C12—N2—C16	0.6 (4)
N1—C5—C9—O1	−1.2 (3)	C15—C16—N2—C12	2.4 (3)
C4—C5—C9—O1	179.6 (2)	C20—C16—N2—C12	−176.7 (2)
N2—C12—C13—C14	−2.4 (4)	C8—C9—O1—C10	−3.9 (4)
C12—C13—C14—C15	1.1 (4)	C5—C9—O1—C10	176.3 (2)
C13—C14—C15—C16	1.6 (4)	C11—C10—O1—C9	−169.7 (3)
C13—C14—C15—C17	179.3 (3)	C19—C20—O2—C21	−7.9 (3)
C14—C15—C16—N2	−3.5 (3)	C16—C20—O2—C21	171.53 (19)
C17—C15—C16—N2	178.8 (2)	C22—C21—O2—C20	−167.1 (2)
C14—C15—C16—C20	175.6 (2)	O1—C10—C11—F3	59.6 (8)
C17—C15—C16—C20	−2.2 (3)	O1—C10—C11—F1	−60.0 (9)
C14—C15—C17—C18	−176.4 (3)	O1—C10—C11—F2	171.3 (6)

### Hydrogen-bond geometry (Å, °)

**Table:**

Cg1, Cg2 and Cg3 are the midpoints of the N1–C5, C5–C9 and C17–C18 bonds, respectively.

**Table:**

*D*—H···*A*	*D*—H	H···*A*	*D*···*A*	*D*—H···*A*
N1—H1A···N2	0.86	1.87	2.684 (3)	158
C22—F5···*Cg*1	1.33	3.10	3.796 (3)	111
C22—F6···*Cg*2	1.33	3.17	3.804 (4)	109
C13—H13···O3^i^	0.93	2.60	3.393 (6)	144.
C21—H21B···O4^ii^	0.97	2.48	3.437 (7)	169
C22—F5···*Cg*3^iii^	1.33	3.24	3.860 (8)	108

Symmetry codes: (i) −*x*+1, −*y*+1, −*z*+1; (ii) −*x*+1, −*y*+1, −*z*+2; (iii) −*x*+2, −*y*+1, −*z*+2.
